# Diabetes

**Published:** 1879-08

**Authors:** W. W. Hall


### *DIABETES.*

By W. W. Hall, A.M., M.D.

There are two forms of this deadly disease, the *diabetes insipidus* and the *diabetes melletis.* The former ordinarily runs into the latter. The daily secretion of water is frequently enormous, reaching in some cases to five or six gallons. It is a wasting disease, and one which frequently terminates life in a few weeks or months, the sufferer meanwhile eating well, and therefore feeling that nothing special is the matter, when, at the same time, a fatal disease has got hold of him.

The first symptom of this disease which arrests attention, is the frequent call to pass urine, and the fact that a large quantity is voided at each attempt. It is not uncommon for pain to accompany the operation. At the same time there is great thirst, and often a voracious appetite. The mouth is apt to be dry, the tongue clammy and sticky, and often very red; there is flatulence and indigestion, and the bowels are usually constipated.

With the fearful drain on the system which we have indicated, debility and emaciation must surely follow, together with pain, especially in the loins and back. The limbs become feeble and almost powerless, and active exertion is rendered impossible. There is a simple test which those whose evacuations of water is excessive would do well to apply, in order that appropriate remedies may be availed of. It is simply the addition of a tablespoonful of yeast to a pint of the fluid, which must then be stirred and left in a warm but not hot place. If fermentation follows, the presence of sugar is established. Evaporation of another pint will enable the operator to secure the sugar crystals not unlike those of the grape. For a long time there was no known cure for this disease, and its victim usually perished from debility or fell into pulmonary consumption. All attempts to cure the disease by medication have proved unavailing. Nearly every article of the *materia medica* has been tried from time to time, and while there have been occasional instances of amelioration, we have yet to see the first case permanently cured by drugs. Yet, in the present state of our knowledge, we do not look upon diabetes as necessarily a highly dangerous disease. If taken early it can be cured in nine cases out of ten, provided, always, that the patient will submit to be controlled.

Hot baths to induce perspiration are of great value. The truth is, however, that the treatment is chiefly dietetic. The food and drink must be regulated by science, and must be scanned as critically and prepared as carefully as if it were the food of an infant. It must be totally devoid of starch and sugar. The best possible food is the pure Gluten of Wheat freed from white flour, and containing no particles of bran to irritate the internal viscera. This pure, bland Gluten appears to be well nigh specific in its action. It quickly regulates the bowels, and relieves the constipation. It very rapidly nourishes and builds up the system. It restores the brain—which generally suffers in this disease—to a normal condition. The sugar disappears from the urine, and the secretion gradually lessens. When this point is reached, bodily vigor and elasticity will not be long delayed. To obtain the Gluten, address the Health Food Co.

Milk and cream in moderation, and eggs and tender beef and mutton, and such green things as spinach, may be partaken of in all stages of this disease, but the chief article of diet must be the pure Gluten, cooked in any simple way. The fact of the value of Gluten as a remedial food in this terrible disease has been known for forty years. Dr. Camplin of London had diabetes, and relieved it by using Gluten. He thought the relief came from the employment of Wheat bran, and accordingly recommended bran-food for the purpose. Bran-crackers and bran-flour were accordingly prepared for use in such cases, and diabetes was relieved in many instances, and the patient starved to death instead. We have conversed with dozens of victims of the bran theory, some of whom have been too feeble to repeat the story of their misery in a tone above a whisper. We have been able to convince them in a few days of the total fallacy of the bran theory, by restoring some of their old strength through the use of pure, branless Gluten and other appropriate means. All the good derived by Dr. Camplin or any other diabetic patient from bran, came from the Gluten which is always consolidated upon the outer chaff or hull of wheat by the weight of the upper mill stone. There is a good deal of Gluten in all such bran, but it is inseparably associated with the hull, and the hulls when swallowed ruin the digestion. A tea made of clean, fresh bran would do good service, were it not that to drink it to any useful extent would necessitate swallowing more fluid than a diabetic patient should take. For drinks in this disease only Gluten tea, or pure water, or Cereal Coffee, or milk, or buttermilk are admissable. As we have said, very little fluid of any kind should be taken, the thirst being properly quenched with crumbs of ice. Ale, beers and wines, and liquors containing alchohol must be scrupulously avoided, as if any of these are used, there is no possible cure. There are other very important regulations to be adhered to, which can more properly be communicated by letter, than made public in type. Sufferers from this or other disease, are privileged to address the Health Food Company with stamp to pay postage on the reply.

The great difficulty in treating this disease is, to induce the patient to do precisely as he is directed. He obeys implicitly for a few days, and, finding his worst symptoms abated, concludes that his case wasn't a very severe one, after all, and plunges into white flour bread, or potatoes, or rice-pudding, or beer, or some other form of dissipation, and suddenly discovers that the old enemy is upon him again, harder than ever to shake off.

